# The bumpy trajectory of performance-based financing for healthcare in Sierra Leone: agency, structure and frames shaping the policy process

**DOI:** 10.1186/s12992-018-0417-y

**Published:** 2018-10-20

**Authors:** Maria Paola Bertone, Haja Wurie, Mohamed Samai, Sophie Witter

**Affiliations:** 1 ReBUILD & Institute for Global Health and Development (IGHD), Queen Margaret University, Edinburgh, UK; 2 ReBUILD & College of Medicine and Allied Health Sciences (COMAHS), Freetown, Sierra Leone

**Keywords:** Performance based financing, Policy analysis, Political economy analysis, Interpretive policy analysis, Framing theory, Sierra Leone

## Abstract

**Background:**

As performance-based financing (PBF) has been increasingly implemented in low-income countries, a growing literature has developed, assessing its effectiveness and, more recently, focussing on the political dynamics of PBF introduction and implementation. This study contributes to the latter body of literature by exploring decision-making processes on PBF in Sierra Leone during the 2010–2017 period. Sierra Leone presents an interesting case because of the ‘start-stop-start’ trajectory of PBF.

**Methods:**

The qualitative case study is based on a document review and 25 key informant interviews with national stakeholders and international actors. Documents and interviews were analysed based on a political economy framework focusing on *actors* and *structure*, but also making use of concepts drawn from interpretive policy analysis to look at *frames*.

**Results:**

Our analysis describes the process of negotiation and re-negotiation of PBF in Sierra Leone, highlighting the role of different players, both internal and external, their ideas, capacity and power relations, and the shifting narratives around PBF. It is shown that external actors driving the debate make use of ‘frames’, both actual (i.e., defining the timing and pace of the discussions, the funding available, etc.) and metaphorical (i.e., how PBF is interpreted, defined and understood) to fit in and influence the debate. This is facilitated by the lack of capacity and resources in the fragile setting. Other strategies, such as ‘venue shopping’ are employed, though they may add to fragmentation in the volatile context.

**Conclusions:**

The retrospective view of the study has an analytical advantage, but findings are also relevant to guide practice. Although power relations and rent-seeking issues are difficult to overcome in resource and capacity-constrained settings, more attention could be paid to other elements. In particular, adopting shared frames to ensure a common and inclusive understanding of technical concepts such as PBF may be useful to ensure the political sustainability of reforms. Also, the ‘actual frames’ which define negotiation and implementation should remain flexible, allowing for disrupting events (e.g., the Ebola epidemic in Sierra Leone) as well as for time to develop national capacity and ownership in order to ensure longer-term political support and better health system integration.

## Background

Performance based financing (PBF) programmes are increasingly implemented in many low income countries and in particular in sub-Saharan Africa, including in fragile settings [1, 2]. Although models and terminology slightly change from one setting to another, a common understanding of the core features and design of PBF programmes has emerged and has been codified [1, 3]. The World Bank has particularly contributed to this process, and is also the major funder of PBF programmes, largely through the Health Results Innovation Trust Fund (HRITF), funded by the British and Norwegian governments [4].

Despite the rapid growth, the pattern of diffusion of PBF in low income settings has not been without shifts and setbacks, both theoretical and operational. The theoretical understanding of PBF has evolved over time, moving from a focus on health workers’ motivation and provider payment mechanisms, with an emphasis on performance (as evidenced, for example, in the name of the Trust Fund) to a more comprehensive understanding of PBF as (an element of) a broader health system reform [5] and as a potential entry point for strategic purchasing [6]. At the same time, critiques of PBF have emerged focusing both on specific aspects, such as the potential for crowding out of intrinsic motivation [7, 8] as well as more broadly on the lack of impact, high costs, and weak theory of change [9–11]. Witter [12] identified different ‘effects’ that PBF may be building on, and on which different actors may be interested to focus their attention on their understanding and description of PBF. These include:

- *Incentive effect* – modifying health workers and managers’ behaviour through rewards and sanctions; increased focus on results;
- *Income effect* – getting resources to the front-line;
- *Autonomy effect* – greater freedom to manage resources at the front-line;
- *Accountability effect* – clearer roles and responsibilities; increased transparency through use of data; better evidence on what facilities and health workers are doing;
- *Intrinsic rewards effect* – better management support and supervision; constructive feedback and support.

At the operational level, recent research has documented the policy processes and the trajectory of PBF adoption and implementation in low-income settings [2, 13–15]. These studies have highlighted different patterns in each context, with some countries able to scale up PBF nationally such as Rwanda and Burundi [16]. Others have successfully transitioned from external to national implementation agencies as in Cameroon [17], and in others PBF was discontinued after piloting as in Chad [18].

The present study adds to this growing but still limited literature by presenting the case of Sierra Leone. It explores the policy and decision-making processes on PBF in a retrospective manner covering the post-conflict, Ebola and post-Ebola periods, from 2010 to the end of 2017. The experience of Sierra Leone has not been documented so far. However, it represents a particularly useful and relevant case study because PBF was implemented at national scale (one of the few countries in sub-Saharan Africa) and also because of the “start-stop-start again” trajectory that PBF has followed over time. Our analysis examines the role of the drivers of the policy process, including national and external actors, their interests, agendas, and dynamics between them, in interaction with the features of the context. However, it also explores how the evolving framing and understanding of a technical concept such as PBF, driven by the global evolution in PBF's theoretical understanding but also by local dynamics, has influenced the policy trajectory. The aim of the study is to unpack what has happened in Sierra Leone concerning PBF, how and why. We build on these findings to reflect on the role and the current practices of external organisations, and how they could be shaped to support policy processes in ways that allow building consensus, ensuring national ownership and promoting the long-term political sustainability of reforms, in particular in (institutionally) fragile settings.

## Methods

The research is based on a qualitative case study which draws on a document review and a series of key informant interviews.

Documents collected are mostly grey literature and policy documents relating to PBF (e.g., operational manuals, presentations, reports, PBF reviews, blogs, etc.) and to the broader health system in Sierra Leone (e.g. health system strategies, evaluations and reviews, situation analyses, policies on human resources for health and maternal, reproductive and child health, health priorities and recovery plans, meeting reports, etc.). They were collected through our involvement in country over the last years, but also by asking key informants to provide the documents they mentioned during the interviews. Additionally, targeted internet searches on relevant websites (e.g., World Bank's RBF website, WHO, Cordaid) were also carried out. In total, 68 documents were retrieved.

Key informant interviews were carried out in November 2017 in Freetown or via Skype for those abroad. Ethics approval for primary data collection was obtained from Queen Margaret University’s Ethics Board, as well as the Sierra Leone Ethics and Scientific Review Committee. Interviewees were purposefully selected among the stakeholders involved in PBF in Sierra Leone with the aim to be as comprehensive as possible. Key staff in the relevant bodies working on PBF or involved in the debates around it were identified building on previous work and contacts in country, as well as by asking interviewees to suggest others. Potential respondents were contacted via email or telephone or in person during meetings. Because the interviews took place in Freetown, sub-national actors are underrepresented in this sample. Additionally, a few of those contacted, in particular among the international actors, did not reply and their views were not included in this analysis. In total, 25 key informant interview were carried out with government staff at central level from the Ministry of Health and Sanitation (MoHS) and other relevant public bodies (*n* = 9), MoHS staff at district level (*n* = 4), donors and NGOs (*n* = 7), and technical advisers (TAs) both to donors and to the MoHS (*n* = 5). Interviews were recorded and transcribed verbatim for analysis. Additionally, we draw on the findings from previous studies [19–21], and re-analysis of interviews carried out for those studies in March and September–November 2013 and April 2014, which included the national level but also district and facility levels. Given the involvement of all co-authors in the policy landscape in Sierra Leone since around 2012, direct observation of the dynamics concerning health financing, human resources for health and PBF, for example, by participating in meetings between the MoHS and development partners or in the discussions during in-country dissemination of our previous findings, also provided a source of information for this study.

The data collected and analysed (or re-analysed) in this work focused on the processes that led to the adoption, implementation, discontinuation and then re-introduction of PBF in a retrospective way covering the period from 2010 to 2017, to investigate not only what had happened, but also how and why.

**Fig. 1:**
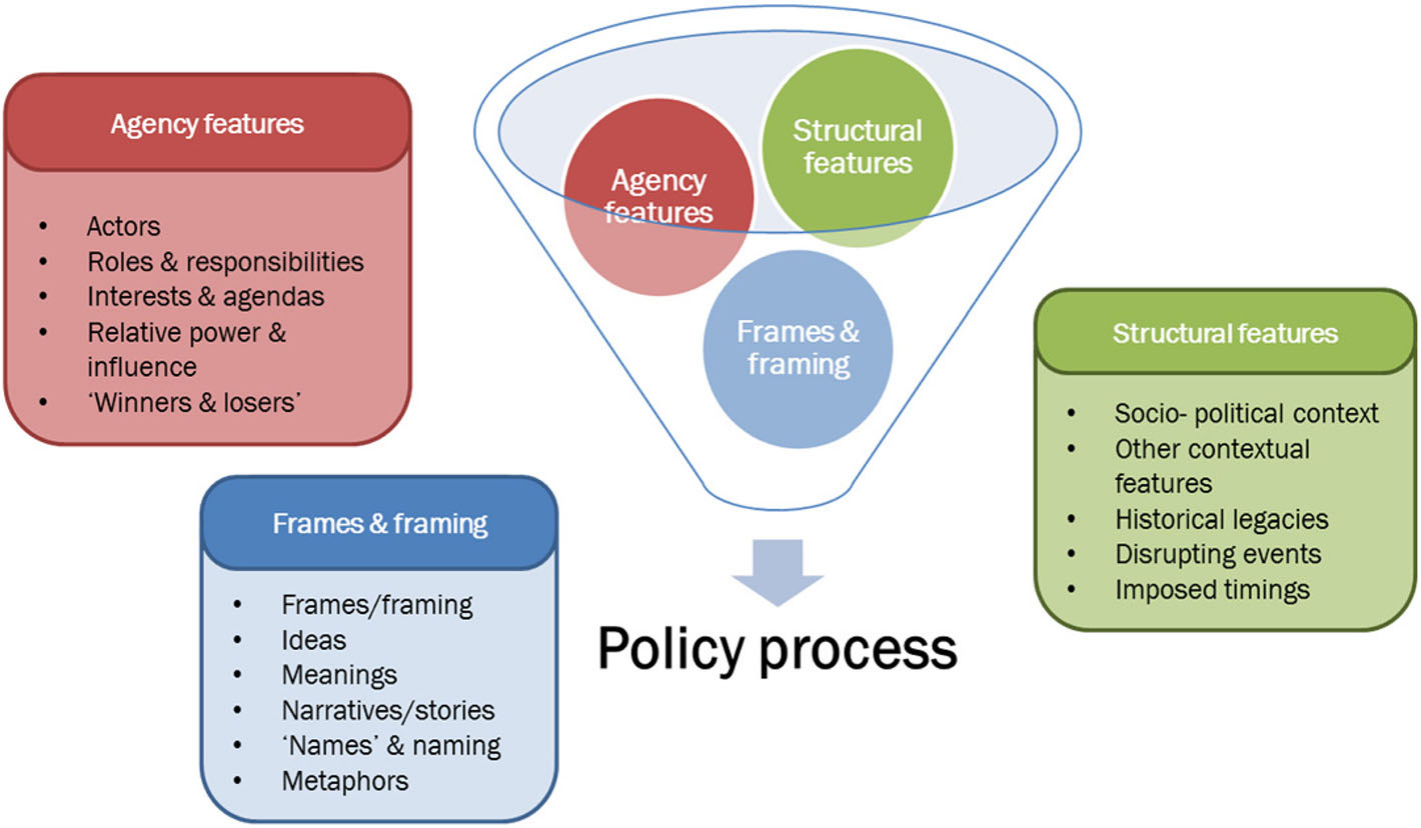
Analytical framework for the analysis of the policy process on PBF in Sierra Leone, looking at the interplay of agency, structure and frames

Data analysis of both oral and written sources takes a political economy approach, looking at the dynamic and evolving interaction between *actors*, their interest, agendas, relative power and influence, perceptions on PBF design and implementation (including success, challenges, changed roles and responsibilities, compensation for those changes, impact on rent-seeking, etc.) with the broader *structure* (historical legacies, political, economic and epidemiological context, key events, etc.) [22]. However, going beyond the traditional political economy approach and following Cairney’s ‘complementary’ approach [23] to combine insights from multiple theories to explain a case, our analytical framework also adopts concepts and perspectives drawn from interpretive policy analysis [24] and in particular framing theory and frame-critical analysis [25]. This allows us to include *frames* (alongside agency and structure) into the picture of the variables which interact to shape the policy process (Fig. 1) and to observe how frames, ideas, meanings, narratives and names (in our case, of the ‘technical’ concept of PBF) are constructed and co-constructed among actors during the agenda setting and policy development processes. A recent review of the literature on framing in health policy [26] has identified a nascent scholarship and highlighted the potential value of constructivist and interpretative approaches for health policy analysis, therefore confirming the importance of adopting framing, as theory and as methods, to gather insights into the forces shaping political debates in the healthcare arena.

Data were analysed using a framework approach [27], based on a list of pre-developed codes which reflected the main elements of the analytical framework (Fig. 1). Because our retrospective approach covers a long period of time (2010–2017) during which PBF was debated, designed, implemented and again debated in Sierra Leone, in order to be comprehensible to the reader, the ‘results’ section is initially organised in chronological order to detail the more than half decade-long policy process. We then focus on the elements of the analytical framework to explain how they acted and interacted to influence the policy process. Anonymised quotes are provided. To preserve anonymity, respondents are described only as “governmental” (national and district level actors, and TAs seconded to government) and “non-governmental” actors (donor/NGO staff and TAs/consultants working directly with them).

## Results

### The policy process: Decision-making on PBF (2010–2017)

In this section, we provide a description of the policy processes that underlie the adoption, design and implementation of PBF since 2010. Our analysis suggests that the longer-term policy making process can be divided into four key periods.

#### Introduction of the concept of PBF in Sierra Leone (2010–2011)

In September 2009, the then President of Sierra Leone announced the launch of the Free Health Care Initiative (FHCI), which introduced fee exemptions for pregnant women, lactating mothers and children under five. The FHCI represented a key turning point in the health sector in Sierra Leone. It sparked a fervent, if rushed, process to prepare its implementation (which started in April 2010) and to design a series of corollary policy measures. An earlier analysis of that process [19] found that, during the FHCI preparation, the idea of introducing PBF (alongside providing funding for it) was brought to the table by the World Bank.^1^ This was done through the Human Resources for Health Technical Working Group (HRH TWG), led by the HRH Directorate of the MoHS, which was in charge of coordinating the FHCI-related reforms associated with HRH. The introduction of PBF was seen as one of the options to compensate for loss of (formal and informal) income of facilities, increase health workers’ income and motivate them to deal with the expected increase in workload after the FHCI reform. The alternative option, which was technically and financially supported by a different donor, was that of a blanket salary increase for all health workers, coupled with in-kind provision of FHCI-related drugs. Ultimately, the latter option was agreed on and, while the conflicting agendas and ideologies of the two donors involved in the debate undoubtedly played a role, the choice was justified on the basis of practical feasibility considerations since a salary increase would take less time to implement and entail lower transaction costs than PBF.

At this point, the discussion on PBF became detached from the FHCI planning and from the HRH TWG and the HRH Directorate, which remained in charge of the implementation of the salary increase and related reforms (including managing the payroll and the health workers’ “Attendance Monitoring System” and “Staff Sanctions Framework”). However, meetings on the PBF design continued between the World Bank and the Directorate for Policy, Planning and Information (DPPI) of the MoHS [19]. In the prevailing narratives, PBF was meant to contribute to the motivation of health workers especially in terms of quality of service and partially compensate for the facilities’ loss of income, while the salary supplementation was seen as compensating the extra workload and the drug provision to reduce the need to charge fees. The performance focus was in line with the broader Presidential agenda at the time which also stressed the idea of performance contracting and monitoring at all levels of the government, for example with the signature of Performance Management Contracts for senior managers at ministerial level [28]. However, these contracts were rarely enforced and the envisaged Individual Performance Appraisal System (IPAS) for lower-level staff was never implemented (it was again being discussed at the end of 2017 – direct observation).

PBF was strongly supported by the World Bank and was designed with its financial and technical aid over the following year. The design envisaged PBF to be introduced nationwide without piloting and making use of existing structures. This included using public facilities for the provision of services, District Health Medical Teams (DHMTs) in collaboration with Local Councils for verification, supervision and data management at district level, the Health Financing Unit (HFU) of the DPPI for the implementation of the PBF reporting and financing system and the Local Government Finance Department of the Ministry of Finance and Economic Development (MoFED) for facility payment [29]. The collaboration with the Local Councils stemmed from the fact that they are -in theory- responsible for the provision of healthcare services under the Local Government Act of 2004. The PBF scheme, which initially included only primary facilities^b^ and only six reproductive, maternal and child health services, was named “Simple Performance-Based Financing Scheme for Primary Healthcare”. According to the operational manual, the aim was to implement the ‘simple’ scheme during a first phase from 2011 to 2013, to then build on the lessons learned to “expand to a Comprehensive PBF Scheme that covers both primary and secondary healthcare services at a later date” ([29]: p.viii). The implementation of the PBF scheme started in April 2011.

#### Implementation of the national PBF scheme for primary care (2011–2016)

After its inception, the implementation of PBF continued with no major revisions to the initial arrangements. For example, the plan envisaged in the Operational Manual to contract an agency (NGO or CSO/Civil Society Organisation) to provide frequent, independent validation of PBF performance reports was never put in place. In addition, a number of challenges started to emerge and to be informally discussed among stakeholders in the health sector (mainly, DPPI, DHMTs, NGOs supporting primary facilities, and health workers), but were rarely if ever systematically reported.

In March 2013, a corruption scandal emerged concerning the misappropriation of GAVI funds by some MoHS staff within the DPPI. Twenty-nine people were formally accused of the crime, lost their jobs [30], though they were eventually exonerated later on. The scandal had the unfortunate consequence of leaving the DPPI and in particular its HFU practically emptied of its staff, with a loss of institutional memory, operational capacity and skills. Only relatively junior staff, mostly externally paid rather than civil servants and in very low numbers (1–2 people at times) remained and were in charge of running the PBF scheme. In the process and also because the dwindling funding, the original idea to scale up the ‘simple’ scheme to a ‘comprehensive’ one in 2013 was abandoned and, despite the efforts to review the implementation and take stock of lessons learned, for example, in workshops organised in June–October 2013 [31, 32], many challenges remained.

In April 2014, the first PBF external verification was conducted as planned and the report released [33], by the Dutch NGO Cordaid, an organisation with well-established expertise in PBF. One of the key informants called it an “eye opener” (KII–government actor) as the report identified a number of fundamental problems, such as: the considerable difference in number of services provided and in quality scores between the internal and external verification; the weaknesses in financial management; the extremely long (up to 1 year) delays in payment of the PBF bonus; the weak institutional arrangements that did not allow for a clear separation of functions (for example, with the DHMT being at the same time the technical supervisor of facilities and the verifier); and the insufficient involvement of the Local Councils which in practice were little engaged and rarely participated in PBF implementation. With reference to the weak institutional organisation, the report states that “in Sierra Leone, a ‘light’ PBF approach is applied” ([33]:p.69). The review also highlighted positive aspects of the PBF programme, such as that it had allowed facilities to directly purchase items to improve the work environment (more hygiene, better equipped buildings and better supplies), thus exuding a more nuanced and complex understanding of PBF not only as a way to provide cash to facilities, but also to allow increased management autonomy.

The elements (both positive and negative) identified in the external verification are echoed in the interviews carried out at central and district level for this research, and are similar to the views of health workers captured in earlier studies [21, 34]. In particular, respondents stressed that, despite the fact that PBF made a difference by providing extra cash at a time when “fund flow was limited from the MoHS” (KII-government actor), a number of implementation challenges limited its impact. These included weak financial and managerial procedures and lack of transparency, both in sharing the payment between staff and in using it, as well as possible corrupt practices at all levels (including central, district and facility), issues related to the challenges in the banking system (e.g., lack of facilities’ accounts, wrong bank details, general weakness of the banking system, errors in entering information, etc.) and delays in payment. The latter were seen as demotivating and delinking payment from performance. This impression was compounded by the widespread perception of the PBF payment as a sort of salary (rather than an incentive) for the majority of health staff, in particular for the unsalaried volunteers [35]. Additionally, many staff at facility but also at district level appeared not aware about how the payment was calculated [36]. Some interviewees also noted that very limited data, analyses, information and research were available on PBF and its effectiveness, beyond views and perceptions. While many PBF programmes have been subject to rigorous impact evaluations [4], the scheme in Sierra Leone did not have such mechanisms so that extremely limited evidence exists, based on the external reviews [33, 35], a one-off analysis carried out internally [37], and a qualitative study [21].

During the Ebola Virus Disease (EVD) outbreak in 2014–2015, PBF continued to function in the sense that payments were made to providers based on the monthly HMIS report, but the internal verification was suspended since DHMTs could not visit facilities ([38]; KIIs). However, extreme delays in payment and other implementation challenges affecting the programme continued. Additionally, around 6 million USD from PBF funds were reallocated by the World Bank to the EVD emergency response (KIIs), which accelerated the impending end of the project.

#### Design, implementation and discontinuation of PBF PLUS (2015)

Building on their involvement in the external review, Cordaid with the MoHS started planning the launch of a new (pilot) PBF scheme around 2014. Based on their own definition of the national PBF as “light PBF” ([39]:p.18), the new programme was named “PBF PLUS” and its aim was to propose a new design to address the challenges identified in (what became known as) “PBF Light”. In order to do so, PBF PLUS envisaged more complex institutional arrangements, for example with the creation of a “Performance Purchasing Assistance Team” at district level in charge of verification, coaching of facilities, and assistance in the preparation of facilities’ business plans. The project included primary facilities, but also the district hospital and provided payments for more services than PBF Light (18 for primary facilities compared to the previous 6), as well as clearer incentives and indicators for DHMTs performance [39]. Similarly to NGO-implemented PBF pilots in other settings [2], it also aimed at creating a narrative of PBF success. In particular, in Sierra Leone PBF PLUS was meant to act “as a catalyser” for health system strengthening, in an environment where PBF was increasingly seen as not functional and about to be discontinued. Following participation in an international PBF course which highlighted the challenges of the ongoing programme and despite the EVD epidemic, PBF PLUS was designed in early 2015 and briefly implemented with Dutch funding in Bombali district^c^ from May to October 2015 [39].

Although judged successful by the implementers [40], PBF PLUS lasted for an extremely short period of time. Cordaid had hoped to raise profile and funding by shifting the narrative to that of a ‘successful’ PBF, also in light of the upcoming renegotiation of the World Bank-funded national PBF (see below). It was hoped to influence the political dialogue at the highest levels and muster the support of “PBF champions” at the MoHS and the MoFED (the office doors of many key actors involved in PBF PLUS have a sticker identifying them as part of the “PBF family”). However, this did not work as planned and Cordaid lost some of their political clout over time (KIIs). Additionally -or perhaps as a consequence- an alternative narrative about PBF PLUS emerged which was repeated by many respondents, and focused on the fact that PBF PLUS’ implementation at about 2 USD per capita in subsidies only [40] was too costly compared to the national PBF scheme at 0.4 USD per capita [41] and therefore unsustainable.

“It (PBF PLUS) was nicely set up, but not sustainable because it was too expensive and also, they (Cordaid) had lost the … I don’t know if it was personality-related. Because they had such a short time to implement, they pushed a lot of things through the Ministry where the Ministry would have wanted more time, but the time was not there, so I think they lost credibility, and trust and relationship with the Ministry in that process” (KII-government actor).

“It is obvious that there were opposing views in the Ministry between different departments, between individuals. (…) [Cordaid] has been dealing with the PBF unit [HFU], whereas maybe the real decisions were made at a different level” (KII- non-government actor).

As presciently warned in PBF PLUS’ Project Implementation Manual, “a funding gap in 2016 will (have an) effect on the credibility of the health system and PBF in particular” ([39]:p.29).

#### Discontinuation of national PBF and ongoing discussion (2016–2017)

In the meanwhile, the implementation of the national PBF scheme continued up until the end of the World Bank funding in early 2016. Informally, the potential extension of PBF was discussed by some stakeholders, such as WHO [42]. However, these discussions happened in the immediate post-EVD phase, when political priority has shifted to infectious disease control and to the Presidential Recovery Priorities, which did not include health financing and PBF [43]. Additionally, the landscape in the health sector was particularly fragmented given the sudden arrival of many international donors and NGOs with diverging agendas and different degrees of contextual knowledge and understanding. At the time, the prevalent narrative on PBF was stuck between the acknowledged problems of the national scheme and the costs of PBF PLUS framed as high and unsustainable. Evidence was mostly used in a negative way, by stressing the lack of it as justification for discontinuation (direct observation).

Overall, no clear and explicit decision was taken about the discontinuation and/or the future of PBF at the time – a key informant called it a ‘non-decision’ (KII-government actor). The unfavourable timing of the end of the PBF project (in the midst of the post-Ebola recovery) is one possible reason for the lack of renegotiation talks to ensure new funding and avoid PBF discontinuation. Additionally, it appears that the political support for PBF faltered from both the MoHS and the World Bank. From the MoHS side, the views were not unanimous (as discussed below), and while the DPPI was generally supporting the continuation of the programme, others (individuals or departments) were less enthusiastic. One view shared by some informants is that PBF’s challenges, the unclear flow of funds, the complaints and tensions it had created (in particular at hospital level: the hospitals not included in the scheme were vocally complaining while in the pilot tertiary hospitals, staff has threatened to strike because of the way PBF was shared among personnel was perceived as unfair) had made PBF politically risky especially in the run-up to the 2018 general elections (KIIs). At the same time, the World Bank had a new team responsible for the health sector in Sierra Leone and (possibly) interested in distancing themselves from the previous project, which was perceived as unsuccessful. As one respondent said, “they’d rather just let it (PBF) die and restart again” (KII-government actor).

Indeed, discussions for the next iteration of PBF started again not much later, around early 2017 and were ongoing at the time of data collection in November 2017 (direct observation). Although details were not yet defined, the focus of the discussion was on the launch of a pilot PBF scheme in two districts under a fully revised design.

### Drivers of the policy process: The interplay of agency, structure and frames

#### Agency: Actors, interests, agendas and power relations

The MoHS certainly was a central actor in the policy process described above, although it is clear that decisions were for a large part driven by external actors. In particular, the MoHS’ ability to lead and drive policy making appears to be limited by two main factors. The first relates to the lack of capacity within the MoHS, the DPPI and in particular the HFU, before but even more starkly after the GAVI scandal, in terms of both number of staff and skill sets, as there are very few health economists or health financing experts in country. Lack of capacity is likely related to the historical legacies (discussed below) of the protracted conflict and socio-economic disruption as well as the low income status of the country, which have hampered the development of specialised academic facilities in the country. The lack of capacity in the public service is compounded by the fact that civil servants are poorly paid and sometimes unpaid, and many prefer other careers. Many respondents discussed the lack of capacity at MoHS level:

“Because of lack of leadership, [and] lack of capacity at central level, everything just crumbled like that” (KII-government actor).

“They don’t have the leadership capacity to run a unit like that. The problem was that kind of capacity is hard to find. (…) They are about to embark on a radically complex set of (health financing) reforms at the same time, and there is literally no one in this country, no one who understands how to put all these things together” (KII- non-government actor).

The second element relates to the fact that the MoHS is rarely a cohesive entity, and views, interests and agendas, but also political weight and access to resources and power, differ between directorates, programmes and individuals. The MoHS often seems to work in silos and conduct “parallel conversations” (KII- non-government actor) based on bilateral relations between a programme or directorate and a specific donor. Some suggested that, in the context of extremely scarce resources (where, for example, some central-level staff work as unsalaried ‘volunteers’) a key driver of many choices relates to rent-seeking opportunities. In this context, the PBF programme (and the potential rent-seeking opportunities related to it) was seen by many as the fiefdom of the DPPI, while others in the MoHS were excluded, in particular because a “central-level PBF” which could have distributed financial incentives more widely was discussed but never took off. Additionally, some key informants mentioned that those managing PBF were seen as quite powerful before the GAVI scandal, and it is possible that in the aftermath of it, a political choice was made to leave it rather weak and devoid of leadership and technical capacity.

“The DPPI was managing (PBF) and kind of keeping it from the rest of the Ministry. (…) There was a lot of money attached to it. It’s unclear if there were kickbacks or what exactly the issues were…” (KII-government actor).

“Even in the past, the design of PBF it seems to happen more or less in isolation, it’s not properly connected to other things, and that might also undermine the support for it because, yes, you know, if you’re not involved or you don’t feel involved, how are you going to support something like that?” (KII- non-government actor).

“PBF was not benefitting people at the helm of things (…), so to some extent for them, the project is not viable” (KII-government actor).

“A story is sometimes suggested that there hasn’t been buy-in from central level into PBF because the central level wasn’t benefitting from it (…). There is this section in the Operational Manual for the central level PBF, and that’s designed to be a payment to central level staff for having operating PBF (…), so yes, the situation is that the government didn’t want to agree because progress wasn’t being made on the central level PBF” (KII-government actor).

“At our centre here, we are going to advocate for us to also receive incentives. We had issues about the indicators so it never took off” (KII-government actor).

At central level, the political landscape goes beyond the MoHS and is further fragmented. In particular, the influence of the President’s decisions and priorities has much shaped policy making in the health sector, in particular at the time of the FHCI [44]. While a ‘performance’ focus has been high on the agenda of the (then) President in the early 2010s (though less in the following years), PBF was only partially framed under and aligned to that agenda. The Presidential priority for health financing was focused on the creation of the Sierra Leone Social Health Insurance (SLeSHI). The discussion on it has been ongoing since 2008 under the leadership of the MoHS, but was put aside during the FHCI reforms (KII – non-government actor). Later on, around 2015, the Ministry of Labour and Social Security was given the lead in the debate and made some progress on the design. SLeSHI was official launched just weeks before the 2018 general elections [45], though the scheme is not yet operational.

“Sierra Leone is very political in that if the President makes it his priority then it’s going to get done, like the free healthcare was his priority, so it’s done. SLeSHI is his drive, like he wants to have it done. PBF is not, it’s driven by donors, maybe the Ministry, so that then just falls under the knife” (KII-government actor).

“The government’s focus is not on PBF. At Presidential level, it’s on the health insurance scheme. The Bank wanted the focus of the government to be on PBF. So there was a disconnect. At the end of the day, [they] were not singing from the same hymn sheet” (KII-government actor).

“There is a huge political polarisation between SLeSHI and PBF, Ministry of Labour and Ministry of Health. There’s a big uncertainty around what is going to happen after elections next year” (KII – non-government actor).

A similar fragmentation was observed within the Ministry of Finance and Economic Development (MoFED). The department involved in PBF was the Local Government Finance Department (LGFD) in charge of making the payments to facilities, and which appeared closely involved in the processes and debates around PBF and supporting it, while other departments were rather absent in the debate.

Sub-national levels played a less prominent role, with their views mostly neglected. Local Councils were supposed to actively engage in both PBF schemes as fund holders and verifiers (the latter only in the nationwide PBF), but were in practice rarely involved and did not see their interest in the scheme (KIIs). This is also because the decentralisation process in Sierra Leone is only partially achieved and in practice Councils have limited oversight of, and limited technical and financial capacity to manage the health sector. At the same time, DHMTs were struggling to fulfil the role within the national PBF scheme, both because of lack of adequate funding and human resources (for example, DHMTs were supposed to verify up to 130 facilities each quarter, on top of their other duties [36]). As a result, they were not totally in support of PBF, although during the interviews some highlighted its importance in terms of providing resources for facilities. Similarly, providers’ views were rarely taken into consideration in the national-level debates. The Sierra Leone Medical and Dental Board has been involved in the policy dialogue about PBF, but is seen by many of the managers and providers in the frontlines as a “social club” (KII – government actor), powerful but not representative of their views.

Among the external actors, the World Bank appeared to be the main driver of PBF’s adoption and implementation, by introducing the concept in Sierra Leone, promoting it and providing technical and financial assistance, in line with its prominent role in supporting and funding PBF at global level, usually through the HRITF. However, the nationwide PBF in Sierra Leone only partially benefitted from HRITF funding, with 5 million USD provided starting from 2013, while starting in 2011 the bulk of the funding for the “Reproductive and Child Health Project – Phase 2” (RCHP2) under which PBF was implemented came from the Africa Catalytic Growth Fund (a trust fund funded by the UK and Spain - 25.7 million USD) and IDA funding (13 million USD) [4]. This may in part explain the lack of assessment (impact evaluations are compulsory for HRITF-funded programmes) and in general the lower level of scrutiny of the project. In addition, the team leaders in charge of the health sector projects for the World Bank changed over the period analysed with a particularly high turnover (our calculation based on direct observation is that there have been at least 4 team leaders over the 2010–2017 period) and none has been permanently based in Freetown. A respondent stressed this, saying,

“You’ve got turnover within the Ministry, but also you’ve got turnover of staff within the World Bank… I don’t know, I wonder to what extent the lessons of RCHP2 have actually been internalised by those who are working on it now” (KII-government actor).

The role of other donors seems to be less prominent. Indeed, apart from the animated debate during the FHCI preparation in which different donors supported a salary supplementation against PBF [19], once PBF had become separate from the immediate FHCI reforms and was discussed bilaterally between World Bank and DPPI, other donors mostly remained bystanders of the design and implementation processes. One of the reasons may again be related to the high turnover among representatives that does not allow for familiarity with the complexities of the health system strengthening activities, how they work and interact at implementation level and the different actors involved. Also, the technical capacity on health financing issues available in country is rather limited even among donors and international organisations. Indeed, no government partner, including the World Bank, had full-time staff based in Sierra Leone who are health financing experts. As an exception, during the post-EVD period, the WHO set up a Health System Strengthening unit within the Sierra Leone country office, which included a health financing specialist and took part in the discussion about the future of PBF when the nationwide programme was about to end. However, the unit was donor-funded and did not last long. By the end of 2017, no staff was in charge solely of health system strengthening or health financing. The absence of health system strengthening specialisation is not unique to Sierra Leone and similar to many WHO country offices [46]. Finally, respondents stressed that donors seem to have a myopic agenda focused on ‘their’ projects, which results in a fragmented landscape of projects only partially coordinated at national level and very disjointed at sub-national level [20].

“They (the donors) are not interested. I think donors also benefit, they can come in with their own little area of intervention they want to do. No one is interested in health financing because they don’t see the benefit of it” (KII-government actor).

“Maybe this chaos is in the interest of the people who have decision-making power now” (KII- non-government actor).

Similarly, with the exception of Cordaid, NGOs did not play a substantial role in the policy dialogue around PBF. This is because most NGOs were not directly involved in the design and implementation of PBF and many remained sceptical about PBF or were openly opposing it on ideological grounds. At local level in districts and facilities, PBF mostly operated in parallel with NGO activities, although a few instances of collaboration were highlighted where NGOs supported DHMTs for verification activities and provided specific coaching to facilities on indicators and practices related to PBF [20].

#### Structure: Contextual features, historical legacies, disrupting events and imposed timing

The actors described above interact among themselves to shape the policy dialogue, but are also influenced by the structure around them, which includes historical legacies, contextual features and specific events and timings. The recent history of Sierra Leone and the prolonged civil war but also the colonial and post-colonial periods left a legacy of poverty and inequality, which in turn have shaped the context in ways which have an influence on policy making [19]. Relevant for us are, in particular, the lack of opportunities for education and even more for specialised education, which lead to weak technical capacity. As a consequence, there is a lack of understanding of technically complex concepts and reforms such as PBF, not only in terms of the nuances of its role within the broader health financing architecture, but also in terms of basic functioning (especially at sub-national level, among DHMTs and PHUs). Another consequence is that the time and skills available for assessment and evaluation of policies and reforms are limited, so that data and evidence are scarce or absent.

Another relevant contextual feature is the financial dependency of Sierra Leone on aid funding. The National Health Accounts for 2013 revealed that a quarter (24.4%) of the total health expenditure of 95 USD per capita is contributed by development partners with an additional 7.2% by non-governmental organisations. The bulk of it is composed of out-of-pocket expenditures (61.6%) and only a minimal part is contributed by the government (6.8%) [47].

Lack of capacity, of data and evidence, and (arguably most importantly) of funds have profound effect on the policy processes. The consequence is that Sierra Leone is vulnerable to the influence of those providing funds, which are often tied to the design and implementation of specific reforms.

“The underlying governance mechanisms are completely lacking, right? I mean, the government says ‘yes’ to everybody! (…) Whoever is interest in doing it, they can develop their strategy. I’m not sure how much government ownership… I mean, I wouldn’t even say ownership, just how much interest the government has sometimes in its own sector” (KII- non-government actor).

“You’re running an economy that is always donor driven, which is to say if donors do not provide support in certain areas, nothing happens, you know? So it’s so difficult to have control over your own destiny when you cannot actually provide for yourself and most times, the donors come and they have their own agenda, they have their plans, they can ask you questions but they still have their plans and (…) if your plan does not align with the donor’s plan then it is either you lose it or you find a way to aligning your plan to that donor, because, as they say, who pays the piper calls for the tune a lot of the time” (KII-government actor).

In this context, disrupting events both at small scale (such as removal of experienced staff in the aftermath of the GAVI scandal) or at very large, national scale (such as the tragedy of the EVD outbreak) are likely to have major knock-on effects in the already fragile environment.

In the PBF policy process described above, Sierra Leonean institutions had not only limited capacity and possibility of pushing back or promoting issues, but also of changing the pace and timing of certain decisions. While the launch of the FHCI is one example where the timing of a reform was decided internally, other key moments in PBF decision-making (such as the introduction of PBF PLUS, the debate at the end of the national PBF scheme, and the ongoing redesign) happened at times that are defined by outside dynamics related to donors’ funding cycle and deadline imposed by their aid disbursement modalities. In those cases, the consequences appear to be sub-optimal. The discussion on the future of the first PBF scheme, for example, was carried out in the immediate aftermath of the EVD epidemic when priorities, capacity and funds were absorbed and distracted away from health financing issues. Similarly, the ongoing discussion on the next World Bank-funded PBF is pursued under the frame of the Global Funding Facility (GFF), a recently set up World Bank-managed trust fund [48], which, as pointed out in one interview, surely had a role in restarting the debate around PBF:

“With Sierra Leone becoming a GFF country early in the year, I’m sure that sort of lit a fire as well in terms of what needs to happen around health financing, and there was a sort of increased pressure to get PBF in the context of the GFF also more strengthened, I’d say. (…) The GFF provided an added push for new PBF discussions” (KII- non-government actor).

#### Frames, ideas, meanings, names and narratives

To strengthen our understanding of the policy process on PBF, it is interesting to look beyond agency and structure and their interactions, and also draw attention to the frames, ideas, meanings and narratives that are used to define, understand and talk about a fundamentally technical concept such as PBF.

Our analysis highlights how the framing of PBF has evolved over time. Initially, the narrative around PBF focused around HRH issues and PBF was framed as an extra payment to contribute to the motivation and the performance of health workers (partially in line with the ‘performance’ agenda of the President), with a focus on quality of services, while the salary supplementation included under the FHCI was seen as dealing with extra workload.

“During the designing we thought, [...] we have now increased salaries for our health workers. What that means, we are looking at the quantity of services, but in terms of quality, can we look at the quality aspect of it? […] So […] we talked about the performance-based financing […]” (KII-government actor).

Another perspective started to emerge in the PBF narrative, which links less to the ‘incentive effect’ and more to the ‘income effect’ and the possibility of increasing funding at facility level, taking over the policy of providing cash to facilities, which was short lived and briefly implemented.

“What we did was, before we launched the free healthcare, we brought in the idea of upgrading the facilities. We agree that, ‘ok let’s start giving them what we call “cash to facility”, ok’, and [...] we develop guidelines on how to use that cash to facility basically to upgrade their facilities [...]. Basic things, like toiletries, curtains, you name them. [...] And then we used the “cash for facility” as a window of opportunity for PBF to enter” (KII-government actor).

Overtime, the focus on the incentive effect and on health workers’ performance was gradually lost both because of the shifting agenda and narratives at central level, but also because of the increasing delays in PBF payment, which delinked it from the actual performance, and as it was perceived as a substitute for payment, especially for unsalaried staff.

Later on, through the external evaluation and the introduction of PBF PLUS, Cordaid conveyed a broader understanding of PBF not only in relation to its incentive and income effects, but also in relation to better working conditions, increased management autonomy, transparency and accountability. In the design of PBF PLUS, Cordaid explicitly aimed at framing the technical concept of PBF in a much wider way than previously done in Sierra Leone, stating for example that, “PBF is more than just a financing scheme, it is a whole system strengthening approach. […] A properly designed PBF PLUS scheme will lead to improvements in all areas of the health sector” ([39]: p.5). Even the change in naming of PBF with the existing scheme labelled as ‘Light’ in contrast to the proposed ‘Plus’ represented an explicit attempt to shift the narrative.

“Based on those conclusions (of the external evaluation) the idea came to do a project, a more ‘fully fledged’ implementation type of PBF. (…) Let’s see if the Cordaid approach gives a different experience from [the nationwide] PBF, which will became then the Light versus the PBF PLUS. Those names actually didn’t exist before…” (KII- non-government actor).

However, Cordaid’s attempt to create a new, country-specific ‘success story’ does not appear successful, and in fact the implementation of PBF PLUS was seen by many as the declaration of failure of ‘PBF Light’. As one informant stated, at that time,

“I think, the focus was a lot on the problems rather than the good things about PBF” (KII-government actor).

The lack of rigorous evidence on both PBF projects did not help either in the process of shifting the narrative and creating a new story,

“There was a time when we shall have showcase for PBF. But you cannot showcase when you do not have a baseline. (…) What I’m saying is, if you have a baseline, then you will be able to tell a story” (KII-government actor).

During the debate on the discontinuation (or not) of PBF and in the discussions as of the end of 2017 on a new model, some external actors had adopted a broader understanding of PBF, more closely linked to health financing strategies and strategic purchasing and started framing it in that way (see for example, WHO [42]). However, the prevailing way in which PBF was understood, especially by MoHS staff and health workers, still focused on the cash it provided to facilities and the payment for health workers, unrelated to performance. Additionally, very few or no links were made to other aspects of PBF, such as the increased autonomy and accountability and as an integral element of the wider health financing architecture, so that the new discourse was grounded in old narratives narrowly related to increased income for staff and facilities.

“(PBF) is a form of motivational scheme” (KII-government actor).

“I really liked (PBF) because for me it was basically sending around a lot of money across the country” (KII-government actor).

“I would describe (PBF’s understanding) as motivation and as the only source of money at health facility. Over and over again we hear that. (…) Some people are big on the incentives and some people are big on there needs to be money to invest in the facilities” (KII- non-government actor).

“A lot of people just see it as some extra money for the staff. And also people think they are entitled to it, kind of. (…) So it gets completely turned, people even think that this is kind of a salary, but it’s not” (KII- non-government actor).

Although the World Bank team describes PBF as a piece of a complex picture and, in the discussions around the new PBF scheme, has actively promoted and supported a dialogue with Ministry of Labour and Social Security’s unit working on SLeSHI (direct observation at meeting – November 2017), other parts of the health financing puzzle, such as the free healthcare, are excluded from the narrative, at least in the way it is framed for the benefit of the local counterparts. As one respondent said,

“PBF is linked to the FHCI. I mean, *we don’t talk about it in that way*. But the fact that facilities do not charge a sizable number of clients, means that PBF has to be built to accommodate that” (KII- non-government actor, emphasis added).

## Discussion

The study faced some limitations in terms of data collection and analysis. Concerning the first, we recognise that our sample of key informants is biased towards the central level. Although efforts have been made to include DHMT actors in the most recent round of interviews, the sub-national levels are not well represented and the information from NGOs, Local Councils, facility staff refers to previous rounds of data collection. Additionally, our sample is focused mostly on governmental actors, and there are fewer international partners included, reflecting the difficulties in engaging with some of the donors around issues concerning health financing in Sierra Leone, as well as the lack of in-country technical expertise. In terms of analysis, our position as ‘insiders’ within the health financing debates in Sierra Leone (though some authors are positioned more closely to the study setting than others) bears both advantages and disadvantages. On the one hand, as insiders we were able to access the political environment and key actors, and ask meaningful questions on sensitive issues. However, on the other hand, we may have some inherent bias and prejudices arising from the close connection with the research subject [49]. To mitigate the potential limitations, throughout the research process, we attempted to be explicit and systematic in the analysis process, actively reflecting on our positionality and discussing findings and analysis between authors.

Our analysis of the policy process through the lens of actors’ relations, contextual and structural features, frames and framing points to a number of key factors that affected the trajectory. One of the emerging driving factors is the strong influence of external actors, and in particular projects’ funders, which -we found- is exercised across the elements of the analytical framework. This finding is in line with the existing research on the adoption and implementation of PBF in low-income countries, which recognises a common feature in the strong influence of donors, and in particular of the World Bank, in a number of countries, including Tanzania, Cameroon and Chad [2, 13–15, 18, 50, 51]. The case of Chad is particularly relevant as it shares with Sierra Leone the start-stop nature of PBF implementation. There, a recent study [18] stressed how the high turnover of staff at MoH and donors, the top-down nature of the introduction of PBF championed by the World Bank, the non-inclusive implementation processes, and the lack of technical skills at national level resulted in weak ownership of PBF and eventually its discontinuation, despite funding being available. Other studies on PBF and other health financing reforms [13, 52] unpacked the routes of influence that donors utilise and pointed to the control of financial resources and of technical expertise as critical mechanisms and stressed the unequal power that underlie the relations between national governments and external donors. In particular, the World Bank plays an important role as “[it] has a comparative advantage over WHO given its access to ministries of finance, its staff expertise in measurement, its broad multisectorial portfolio and its lending power” ([53]: p.1). The latter, in particular, allows the Bank to back its ideas about health projects and policies with resources for implementation [54]. The downside of this is that the balance of power is easily skewed in the Bank’s favour to the detriment of national ownership and leadership.

In Sierra Leone, we highlighted similar external influences in the PBF policy process. Additionally, dynamics internal to the MoHS also played a role. Indeed, within the resource-strapped environment of the MoHS, rent-seeking was reported by a few KI as a driver of interest and support in PBF, so that those directly involved in the project (DPPI, LGFD/MoFED) and potentially gaining from it in monetary terms or in terms of power and influence, appeared supportive of PBF, while others (other MoHS directorates, hospitals not included in the scheme, etc.) were opposed to it, either genuinely or precisely because of their exclusion. At the same time, donors with little or no incentive to align and coordinate their activities fostered ‘parallel conversations’ with the MoHS. For example, the ‘venue shopping’ strategy [55] adopted -deliberately or not- by the World Bank to move the PBF negotiations bilaterally with the DPPI, once the salary supplementation option had prevailed during the HRH Technical Working Group discussions, proved successful in the immediate term as PBF was designed and implemented. However, it had an unexpected consequence in the volatile context of Sierra Leone. The removal of experienced staff from the DPPI resulted in weakened implementation, created tensions and discontent from other directorates and led to the loss of political support for PBF.

Additionally, our analytical framework is helpful in identifying mechanisms through which the influence is exerted also in relation to the structural elements. Historical and structural legacies shape the context of policy making on health financing in Sierra Leone (including and beyond PBF) in a way that is much defined by the lack of domestic funding and aid dependency. In addition to their influence through financial support, Khan et al. [52] note how donors also control the timing of the availability of resources to run programmes, thus influencing policy implementation and leading to sudden starts and stops in some cases. This is similar to what was observed in Sierra Leone, where ‘actual frames’ (as opposed to the metaphorical frames, discussed below) defined by donors’ funding arrangements and cycles dictated the timing and pace of the policy negotiations, as well as the duration of implementation, and shaped the political space available for influence from external donors. As a consequence, the policy debate is often run at an accelerated pace, which does not allow for the development of national understanding, leadership and ownership over the design of programmes, which others have shown are essential for the ‘institutionalisation phase’ and the long-term political sustainability of PBF [15]. When the externally-driven opening of a window for policy discussion coincides with a (internally) politically sensitive time or a disrupting event, change is unlikely to happen or to be driven by national concerns and dynamics.

Finally, we explored how metaphorical frames, i.e., the narratives, ideas, meanings and even names attached to PBF, have evolved and shaped its trajectory over time. Most evidently, the attempt, through the initiation of PBF PLUS, to create a successful story of effective PBF did not work as planned, and possibly had the opposite effect, because of other factors playing out at the time. As Cordaid recognised, this made it more difficult to avoid the discontinuation of the national PBF once its funding ended. The attempt was not only unsuccessful in terms of creating a new 'success' narrative, but also did not achieve the hoped-for change in the framing and understanding of PBF from a focus on the incentive and income effect to other or all PBF effects. For instance, the emphasis on the potential to enhance transparency and reduce corruption (‘accountability effect’) which is noted as key frame in other settings [13, 15, 56], did not emerge in Sierra Leone, and neither did a broader ‘health system reform’ narrative. Our findings suggest a dissonance in the framing of the same concept. On the one hand, many (national) actors framed PBF focusing on motivation and additional resources, while on the other hand, some (mostly external) actors saw it as a critical piece of the health financing reform – at least in their internal discourse, while they preferred to align the public discourse to the local narrative. Such dissonance in framing may hamper local appropriation and be damaging in the long run. When the conflicting frames will eventually become explicit, they risk blocking further progress in the appropriation and adaptation of the concept and limiting the political sustainability of a reform, which can be perceived as top-down and externally-imposed, as happened in Chad [18].

In the analysis of the framing processes, this study adds to previous work by stressing the role and use of frames not only as rhetorical devices to ‘sell’ the PBF concept [15], but also, in a more interpretive way, as meaning, interpretation and understanding based on individuals’ background, skills, previous experience of it, as well as core beliefs [57, 58]. This is particularly relevant for a concept that is ideologically-charged such as PBF and at the centre of heated debates globally [10, 59].

## Conclusions

In this study, we explored the drivers of the introduction and implementation, discontinuation and possible reintroduction of PBF programmes in Sierra Leone. Our aim was to understand the influence of power relations, incentives, resources and ideas on the trajectory of a very important national health financing policy, such as PBF. We did so by using an adapted political economy framework focusing on the interplay between actors, structures and framing.

The pertinence of our findings points to the relevance of studying and better understanding agenda setting and decision making processes, using qualitative and interpretive approaches. While the retrospective view that we adopt has an analytical advantage, it is important that the lessons learned are given careful attention in a prospective way in order to guide practice. Coordination and alignment between and among different actors (i.e., government at different levels and multiple donors) is certainly no simple task. Power relations and resource issues will be difficult to overcome, in particular in resource- and capacity-constrained settings. However, our analysis suggests that other elements may be possible to deal with and could lead to more positive outcomes. In particular, we stress that adopting broad and shared frames to ensure a common, agreed, inclusive understanding of technical concepts may be useful to ensure the ownership and political sustainability of (health financing and other) reforms. Similarly, in addition to shared metaphorical frames, the ‘actual frames’ which define the negotiation process and the implementation period of projects and reforms should remain flexible, allowing for disrupting events (in particular in crisis-prone contexts), but also for the time and investment necessary for national capacity to develop. In this sense, an unhurried dialogue to co-develop consensual frames may take time (and may not fit with the current structures of funding and negotiation cycles), but could ensure longer-term national ownership, political support and better health system integration.

## Footnotes

1 In fact, the concept of PBF had already begun circulating in a working paper prepared by the Royal Tropical Institute, Amsterdam (KIT) with the local NGO MRC at the time of the FHCI preparation. However, those institutions did not have funding available to support the proposal. ^b^ In April 2012, two tertiary hospitals for maternal and pediatric care were added as a pilot project, with a new set of indicators and incentive calculations. ^c^ At the time, Bombali was EVD-free.
